# Exosome‐Related Gene Signature Predicts Prognosis and Immunotherapy in Gastric Cancer

**DOI:** 10.1155/ijog/1884334

**Published:** 2026-06-19

**Authors:** Qunru Jiao, Rui Zhang, Yingyun Guo, Xiulan Peng, Longshu Zhou

**Affiliations:** ^1^ Department of Rehabilitation, The Second Affiliated Hospital of Jianghan University, Wuhan, Hubei, China; ^2^ Department of Pharmacy, The Second People′s Hospital of Fuyang City, Fuyang, Anhui, China; ^3^ Department of Gastroenterology, Renmin Hospital of Wuhan University, Wuhan, Hubei, China, rmhospital.com; ^4^ Department of Oncology, The Second Affiliated Hospital of Jianghan University, Wuhan, Hubei, China; ^5^ Department of Cardiothoracic Surgery, Sinopharm Dongfeng General Hospital, Hubei University of Medicine, Shiyan, Hubei, China, hbmu.edu.cn

**Keywords:** exosomes, gastric cancer, immune infiltration, nomogram, prognostic signature

## Abstract

Exosomes play a crucial role in tumor progression. However, reliable exosome‐related biomarkers for predicting prognosis in gastric cancer (GC) remain scarce. This study is aimed at developing an exosome‐related gene risk signature (ERGRS) to predict the survival outcomes and immunotherapy sensitivity in GC patients. RNA sequencing data from exoRbase and TCGA datasets were analyzed to identify differentially expressed exosome‐related genes associated with survival in GC. A multivariate Cox regression model was used to construct the ERGRS and a prognostic nomogram. The ERGRS′s predictive performance was assessed using the Kaplan–Meier and receiver operating characteristic (ROC) curve analyses in both training and validation datasets. Furthermore, the relationship between the ERGRS risk score and clinical features, copy number variation, and immunotherapy sensitivity was explored through data mining. Western blotting was performed to validate protein expression levels of key exosome‐related genes in GC tissues. Four exosome‐related genes—TRAF2, ASCL2, NOX4, and MMRN1—were identified as independent prognostic factors. GC Patients with low‐risk scores showed significantly better survival outcomes. Univariate and multivariate Cox regression analyses confirmed the ERGRS as an independent predictor of survival in GC. A prognostic nomogram incorporating risk score, age, and tumor stage was developed to effectively predict patient survival. Immune checkpoint analysis suggested that patients with low‐risk scores may respond better to immunotherapy. The ERGRS represents a promising tool for prognostic prediction and guides the clinical care of GC patients.

## 1. Introduction

Gastric cancer (GC) is a major source of global cancer mortality, with more than 1 million people newly diagnosed worldwide each year [1]. Despite its incidence and mortality having declined over the past five decades worldwide, GC remains the third leading cause of cancer‐related death [2]. Despite advances in novel diagnostic and treatment techniques, the 5‐year survival rate of patients with GC atall stages remains very low (10%) [3]. Whereas the real risk–benefit underlying the use of systemic therapies for each individual is not entirely clear [4], new prognostic markers are necessary to better determine patient prognosis. Hence, the identification of novel biomarkers that provide precise predictive value is urgently needed for improving the prognostication for GC.

Exosomes, ranging from 40 to 160 nm in diameter, are a subtype of extracellular vesicles secreted by most eukaryotic cells. As a key component of the tumor microenvironment (TME), they facilitate intercellular communication by delivering diverse biological molecules [5]. Exosomal components, such as proteins, DNA, mRNA, microRNA, long noncoding RNA, and circular RNA, play a critical role in regulating tumorigenesis, growth, metastasis, and angiogenesis throughout cancer progression [6–8]. Furthermore, exosomes have been demonstrated to promote GC progression and metastasis via multiple signaling pathways [9, 10]. Accumulating evidence indicates that exosomes hold great potential as novel biomarkers for early diagnosis, prognostic prediction, and therapeutic efficacy assessment in GC [11–13]. Nevertheless, there are few published studies that have tried to construct a prognostic prediction model based on exosome‐related genes (ERGs) as far as we know.

In this study, we hypothesized that ERGs could offer prognostic value for GC patients. Using bioinformatic approaches, we constructed and validated an exosome‐related gene risk signature (ERGRS) to predict overall survival (OS) and immunotherapy response. Subsequently, a nomogram was developed to enhance the prognostic performance of the model. As partial experimental validation, exosomes were successfully isolated from clinical samples, and differential protein expression of key ERGs was confirmed between GC tissues and adjacent normal tissues. These findings may provide a useful reference for clinical decision‐making.

## 2. Materials and Methods

### 2.1. Public Data Acquisition and Bioinformatics Analysis

We followed the methods of acquiring ERGs by Guo et al. [14] and Tao et al. [15]. The expression profile data of serum exosomal mRNA, lncRNA, and circRNA sequencing were downloaded from the exoRbase database [16] (http://www.exorbase.org/), including 9 GC patients and 118 healthy individuals. The current exoRbase database represents a significant leap, integrating approximately 3000 samples encompassing both extracellular vesicles and particles (EVPs) and cell‐free RNAs. We downloaded GC transcriptome sequencing data and clinical data from The Cancer Genome Atlas (TCGA) [17] (https://cancergenome.nih.gov/) and the Gene Expression Omnibus (GEO) dataset [18] (http://www.ncbi.nlm.nih.gov/geo/), comprising 373 GC tissues and 32 normal tissues. For the TCGA‐stomach adenocarcinoma (STAD) cohort, single‐nucleotide variation (SNV) and copy number variation (CNV) profiles were also retrieved. The proteomic database of checkpoint response was obtained from Huang et al.′s [19] study. Another proteomic database was employed to explore the immunotherapy benefit from Necchi et al.′s [20] study. Single‐cell RNA sequencing (scRNA‐seq) data were obtained from GSE163558 and analyzed by Seurat and SingleR [21]. The involvement of human subjects from publicly available resources is in accordance with the Declaration of Helsinki. The following clinical information was collected from the TCGA dataset: age, gender, stage, grade, and *H. pylori* infection (Table S1).

### 2.2. Identification of Exosome‐Related Prognostic Candidate Differentially Expressed Genes (DEGs)

We performed differential expression analysis of mRNA expression data from the exoRbase and TCGA databases using the “limma” package (R software v.3.6.1). Exosome‐related DEGs were defined as being significantly up‐ or downregulated when *p* values were < 0.05 and absolute log2 fold change (LFC) > 1. Then, we took the intersection of the DEGs of the two groups. The obtained DEGs were subjected to univariate Cox regression and survival analysis to further screen the prognosis‐related candidate genes. The survival analysis was conducted using the Kaplan–Meier method. Time‐dependent receiver operating characteristic (td‐ROC) analysis was used to predict the prognostic value of the hub genes in the timeROC package of the R package. Gene Ontology (GO) and Kyoto Encyclopedia of Genes and Genomes (KEGG) enrichment analyses were performed on DEGs. Further gene mutation (CNV and SNV) was performed using the Genomic Identification of Significant Targets in Cancer (GISTIC) algorithm.

### 2.3. Construction and Validation ERGRS for Prognosis Prediction

First, in the training set, the univariate Cox regression analysis of OS was carried out to identify DEGs with potential prognostic prediction value. Then, the prognostic ERGRS was built by multivariate Cox regression analysis. We have assessed multicollinearity using the variance inflation factor (VIF). Only when clinical variables with a *p* value less than 0.05 could be selected into the multivariate Cox analysis. Based on the signature, the risk score was constructed in line with the following formula: $\text{risk}\;\text{score}={\sum^{n}}_{i=1}\;\left(\text{coefficien}{\text{t}}_{i}\times \text{expressio}{\text{n}}_{i}\right)$. The risk score formula was derived exclusively from the training cohort in a single fitting procedure. When applied to the validation cohort, we directly used the identical formula and coefficients obtained from the training set for risk calculation, without any refitting or recalibration in the validation cohort. This design ensures an unbiased assessment of the model′s generalization performance. Moreover, all GC patients were separated into low‐ and high‐risk groups based on the median value of the risk score. The prediction accuracy of the signature was evaluated via the td‐ROC curves. To assess the robustness of the ERGRS, we analyzed the correlation relationship between risk score and clinical characteristics using the “limma” and “ggpubr” R packages. In addition, to guarantee the credibility of the results, the prognostic value of the signature was externally verified in the GSE13861 and GSE26901 datasets.

### 2.4. Nomogram Establishment

To further enhance the prognostic utility of the ERGRS, we performed univariate and multivariate Cox analyses on clinical characteristics—including age, gender, tumor stage, and the ERGRS risk score—in the TCGA cohort to identify prognosis‐related clinical indicators. Using the R package “rms,” a nomogram was developed to integrate these predictors. This nomogram assigns a score to each parameter, and the total score corresponds to a single numerical estimate of the probability of a clinical event (e.g., death or recurrence). Finally, calibration curves were plotted to assess the accuracy of the nomogram in predicting patient outcomes.

### 2.5. Identification of Heterogeneous Subpopulations in GC

The R package “Seurat” was employed to process the scRNA‐seq data from the GSE163558 dataset. This included filtering out low‐quality cells, normalizing the data, performing dimensionality reduction, and conducting subsequent analyses. Cancer cell populations were then extracted and reclustered, resulting in the identification of distinct tumor cell subpopulations. Finally, the risk score and the expression levels of ERGs were evaluated across these different cell clusters.

### 2.6. Role of the Risk Score in Forecasting the Effect of Immunotherapy

Heat maps of immune cell infiltration in high‐risk and low‐risk score groups were generated using R programming. To investigate the relationship between risk score and tumor immune microenvironment (TIME), the single‐sample gene set enrichment analysis (ssGSEA) was adopted. ssGSEA calculates a sample‐level enrichment score by converting gene expression rankings into a cumulative statistic. This allows for cross‐sample comparison of specific gene set activity even in the absence of a control group. Second, the expression of various immune checkpoint genes in both high‐ and low‐risk score groups was evaluated. Forty‐eight common targets of immune checkpoint inhibitors (ICIs) were identified, and the associations between the expression of target genes and the risk score were analyzed by utilizing Pearson′s correlation. Finally, the relationship between the immunotherapy response and ERGRS was assessed based on the data cohort of IMvigor210.

### 2.7. Patients and Specimen Collection

The study had been approved by the Ethics Committee of Renmin Hospital of Wuhan University in Hubei Province (No. WDRY2021‐K002). All patients with GC had signed informed consent. A total of four GC tissues and four tumor‐adjacent normal gastric tissues were obtained during surgery at Renmin Hospital of Wuhan University.

### 2.8. Exosome Extraction and Quantification

Exosomes from four paired (GC and normal adjacent tissue) gastric samples were isolated and purified by ultracentrifugation, gently dissociated into small pieces (~2 × 2 × 2 mm), and frozen at −80°C. Briefly, exosomes were separated from tissue using the centrifugation‐based protocol previously described [22], with minor modifications. Tumor pieces were gently sliced into small fragments (1–2 mm) and dissolved in RPMI‐1640 plain medium (Sigma‐Aldrich) for 30 min at 37°C. Subsequently, they were incubated with collagenase D (Roche, Basel, Switzerland) (2 mg/mL) and DNase I (Roche) (40 U/mL). Samples were incubated for 1 h at 37°C in a shaking incubator at 500 rpm. After a filtration step (70 *μ*m pore size), cells and tissue debris were eliminated by centrifugation at 300 × *g* for 10 min and 2000 × *g* for 20 min. Supernatants were centrifuged at 10,000 × *g*avg (Type 45 Ti) for 45 min and 120,000 × *g*avg (Type 45 Ti) for 70 min to collect larger vesicles and smaller vesicles, respectively. All centrifugations were performed at 4°C. Pellets were resuspended in PBS (100–200 *μ*L). Vesicles were further purified by isopycnic centrifugation using an iodixanol gradient (OptiPrepTM, Sigma‐Aldrich). The number and size of exosomes were measured by nanoparticle tracking analysis (NTA) using a Zeta View Particle Metrix (Particle Metrix, PMX‐120, Germany), as previously described [23].

### 2.9. Electron Microscopy

For transmission electron microscopy (TEM) analysis, freshly isolated exosomes were fixed in 1% glutaraldehyde for 2 h at room temperature. Samples were then applied onto carbon‐coated grids, washed with PBS (with the membrane side facing down), and gently dried using filter paper. Negative staining was performed by incubating the grids in 3% phosphotungstic acid (pH 7.0) for 10 min, followed by removal of excess stain with filter paper. Images were acquired using a Hitachi H‐9000 transmission electron microscope operated at 300 kV, equipped with a slow‐scan CCD camera.

### 2.10. Western Blot Analysis

The western blot was consistent with previous methods. Western blot was used to identify the surface markers of exosomes—CD81 (66866‐1‐Ig, Proteintech), CD9 (DL210002), and Tsg101 (DL210005)—and the negative marker protein calnexin (10427‐2‐AP, Proteintech), as well as protein levels of Tumor Necrosis Factor (TNF) Receptor–Associated Factor 2 (TRAF2) (PAB30250, 1:1000, Bioswamp), Ascl2 (PAB47429,1:1000, Bioswamp), Nicotinamide Adenine Dinucleotide Phosphate (NADPH) Oxidase 4 (NOX4) (PAB45612,1:1000, Bioswamp), Multimerin‐1 (MMRN1) (PAB30250, 1:1000, Affinity Biosciences), and *β*‐actin (ab8226, 1:1000, Abcam). The gray value of the bands was obtained using the ImageJ software.

### 2.11. Statistical Analysis

Statistical analyses were conducted using R software (Version 4.1.2) with relevant packages. Data visualization was performed using “ggplot2,” “ComplexHeatmap,” and “clusterProfiler.” Group comparisons between two subgroups or among more than two subgroups were performed using the Wilcoxon test and the Kruskal–Wallis test, respectively. Univariate and multivariate Cox regression analyses were used to identify significant prognostic factors. Survival curves were generated with the Kaplan–Meier method and compared using the log‐rank test. A *p* value < 0.05 was considered statistically significant.

## 3. Results

### 3.1. Identification of Exosome‐Related DEGs in GC

**Figure 1:**
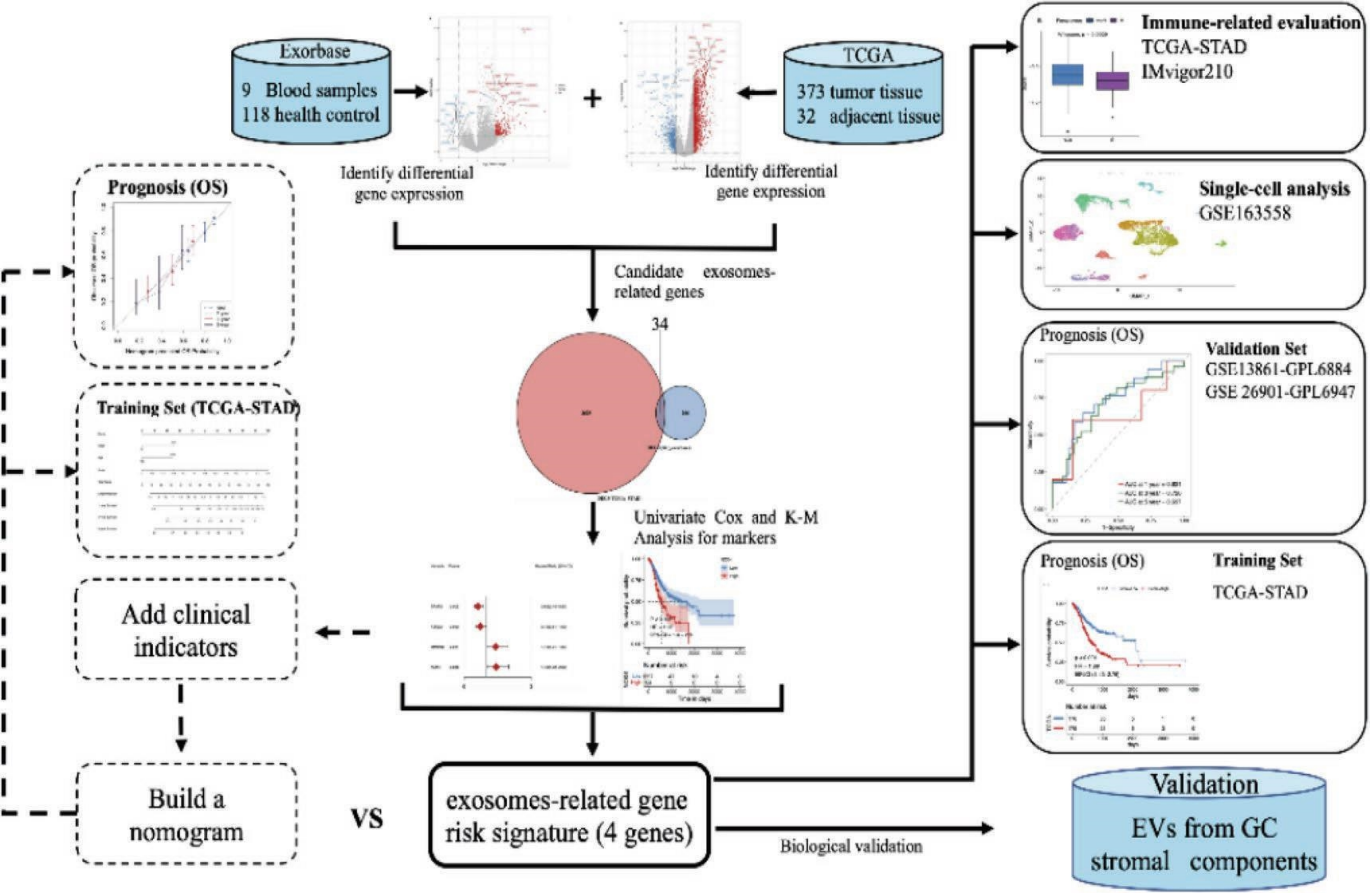
The flowchart of this study.

**Figure 2:**
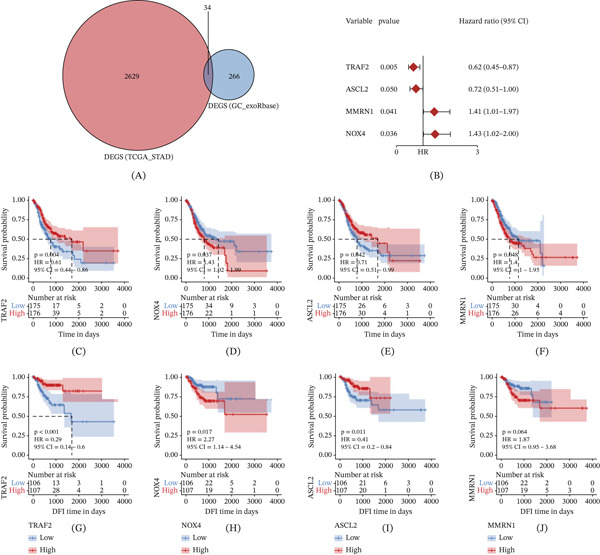
Identification of differential exosome‐related genes. (A) Venn diagram of 34 OCGs. (B) Forest plot of univariate regression analysis. (C–F) Kaplan–Meier′s analysis was used to compare the OS of patients in the four genes (*TRAF2*, *NOX4*, *ASCL2*, and *MMRN1*) of the high‐expression and low‐expression groups. (G–J) Kaplan‐Meier′s analysis was used to compare the disease‐free interval (DFI) events of patients in the four genes of the high‐expression and low‐expression groups.

Figure 1 displays the overall workflow to identify, test, construct, and validate the ERGs of this study. First, 300 DEGs were identified as exosome‐related DEGs based on the exoRbase dataset, using |log2 FC| > 1 and a cut‐off criterion of *p* < 0.05 (Figure S1A). Second, the same steps were executed in the cohort of TCGA‐STAD, and 2663 genes were identified as DEGs (Figure S1B). Third, 34 overlapping candidate genes were singled out through Venn diagrams (Figure 2A) and are listed in Table S2.

To further filter out prognosis‐related genes among DEGs, univariate Cox analysis was utilized, and TRAF2, ASCL2, MMRN1, and NOX4 were identified as key genes related to prognosis (Figure 2B). Survival analyses showed that high expression of TRAF2 and ASCL2 and low expression of NOX4 and MMRN1 were associated with good prognosis: OS and disease‐free intervals (DFIs) of patients (Figure 2C–J). This study further interrogates the biological mechanisms of these four prognosis‐related DEGs in the TCGA cohort. Further observation in GO enrichment analysis showed that these DEGs were mainly enriched in cellular components, including the CD40 receptor complex, NADPH oxidase complex, and perinuclear endoplasmic reticulum. Moreover, they were involved in a molecular function of thioesterase binding and participated in the biological process of positive regulation of protein kinase activity (Figure S2A–C). Moreover, the significant DEGs were correlated to Alzheimer′s disease and pathways of neurodegeneration‐multiple disease in KEGG pathway analysis (Figure S2D).

To interrogate the association between CNV and disease, the gene mutation was further analyzed on 349 overlapping samples of TCGA‐STAD (*n* = 351) and TCGA‐STAD CNV (*n* = 437). As Figure S3A demonstrates, the mutation rate of these three DEGs—MMRNN1, TRAF2, and NOX4—through SNV analysis was 2%–4%. The DEG CNV showed that the mutation rate of TRAF2 (16%), ASCL2 (7%), NOX4 (6%), AND MMRN1 (5%) was relatively higher (Figure S3B).

### 3.2. Establishing and Validating ERGRS

**Figure 3:**
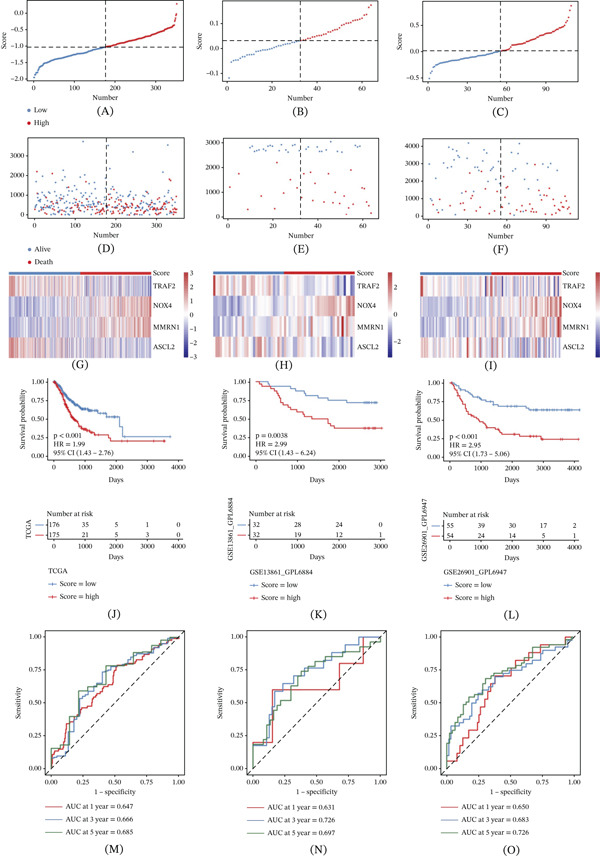
Construction and validation of ERGRS. (A–C) Distribution of risk scores, (D–F) survival status, and (G–I) expression of four prognostic genes in the three datasets. (J–L) Kaplan‐Meier′s analysis was used to compare the OS of patients in the high‐risk and low‐risk groups in three cohorts: (J) TCGA, (K) GSE13861, and (L) GSE26901 cohorts. (M–O) time‐dependent ROC curves of the ERGRS for predicting the risk of death at 1, 3, and 5 years in the three cohorts.

By multivariate Cox regression, TRAF2, ASCL2, NOX4, and MMRN1 were screened, and NOX4 and MMRN1 (hazard ratio [HR] > 1) are referred to as risk genes, while TRAF2 and ASCL2 (HR < 1) are correctly termed protective genes (Figure S4). Then, the corresponding coefficients were obtained to construct the optimal gene signature. The individual‐level risk score of each patient was calculated as follows: risk score (−0.26  ×  expression of TRAF2) + (0.27  ×  expression of NOX4) + (0.078  ×  expression of MMRN1) + (−0.06  × expression of ASCL2). Based on this ERGRS, the risk score was obtained for each individual and was classified into high‐ and low‐risk score subgroups (H and L groups) according to the median risk score of each cohort, respectively. Figure 3A–F shows that the distribution of risk scores and patients′ survival status demonstrates a lower risk of death in the low‐risk subgroup than in the high‐risk subgroup, both in the training (TCGA‐STAD) and validation cohorts (GSE13861 and GSE26901). Besides, different expressions of the four ERGs were observed: NOX4 and MMRN1 were expressed higher in the H group, and TRAF2 and ASCL2 were expressed lower in the H group (Figure 3G–I). In the training and validation set, survival comparison demonstrated that patients in the L group yielded significantly longer OS times (Figure 3J–L). A td‐ROC analysis was applied to assess the time‐dependent accuracy of the ERGRS in prognostic prediction and was validated to have good predictive accuracy in the validation set through AUC values (AUC = 0.647, 0.666, and 0.685, at 1, 3, and 5 years, respectively) (Figure 3M–O). Moreover, the survival analysis comparing the DFI event in the H and L groups showed similar results to OS (Figure S5).

**Figure 4:**
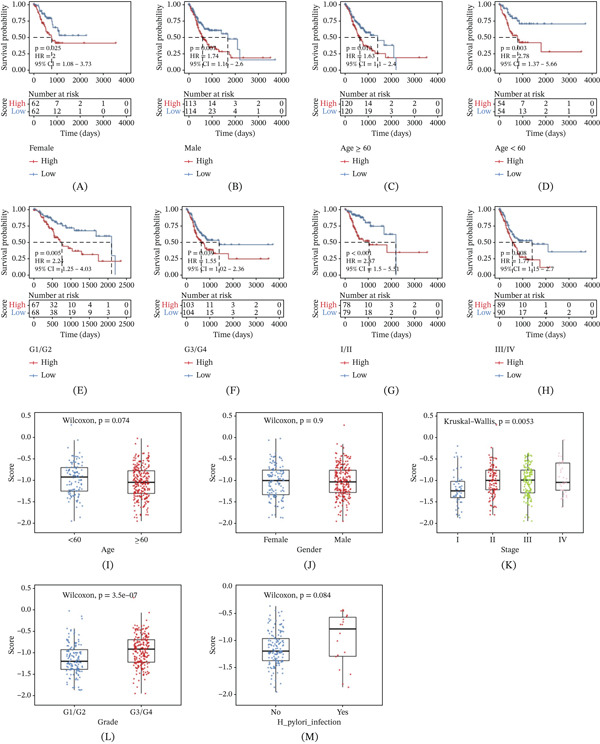
Clinical stratified survival analysis. (A) Survival analysis of male patients. (B) Survival analysis of female patients. (C) Survival analysis of patients with age ≥ 60. (D) Survival analysis of patients with age < 60. (E) Survival analysis of patients with Grades 1 and 2. (F) Survival analysis of patients with Grades 3 and 4. (G) Survival analysis of patients with Stage I–II. (H) Survival analysis of patients with Stage III–IV. Comparison of risk scores in different (I) age, (J) gender, (K) stage, (L) grade, and (M) *H. pylori* infection groups.

Next, to assess the robustness of the ERGRS, the corresponding robustness analysis is presented in Figure 4. We selected different subgroups for repeated survival analysis, and the results showed that patients in the L group have better OS in all clinical subgroups (Figure 4A–H). Similar to the results of robustness analysis, the risk score was calculated for each patient in a different group; the patients in G3/G4 gained a higher risk score than G1/G2 (*p* = 3.5e − 07), and patients in Stage IV gained a significantly higher risk score (*p* = 0.0053) than other stage subgroups (Figure 4I–M).

### 3.3. Establishing a Predictive Nomogram

**Figure 5:**
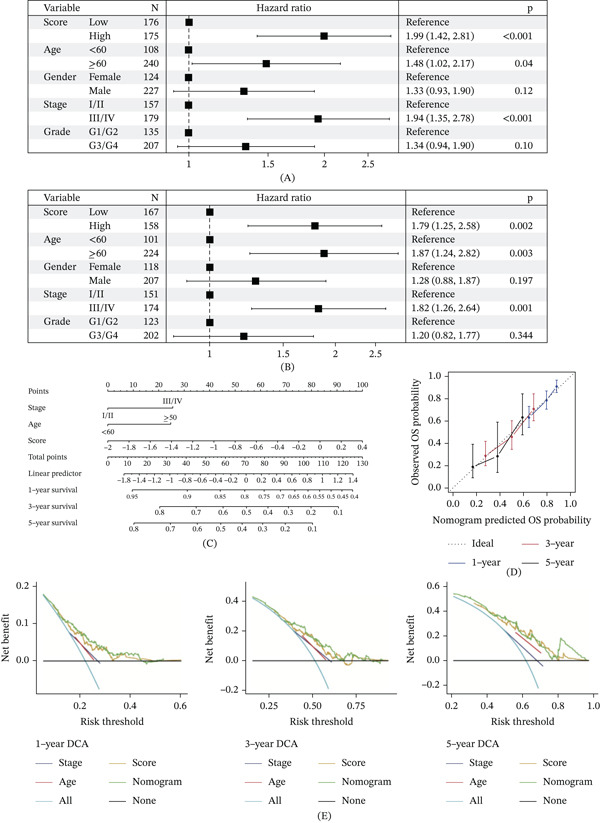
Nomogram construction and calibration plots. (A) Univariate and (B) multivariate Cox regression analyses of clinical characteristics and risk score in the training TCGA cohort. (C) A prognostic nomogram based on age, stage, and risk score to predict 1‐, 3‐, and 5‐year survival probability for patients with GC. (D) Calibration curves of the nomogram for predicting the probability of OS at 1, 2, and 3 years. (E) Decision curve analysis shows the expected net benefits based on the nomogram prediction at different threshold probabilities for the first, second, and third years.

By univariate analysis, we found that risk score (*p* < 0.001, HR = 1.99, 95% confidence interval [CI]: 1.42–2.81), age (*p* = 0.04, HR = 1.48, 95% CI: 1.02–2.17), and stage (*p* < 0.001, HR = 1.94, 95% CI: 1.35–2.78) were linked to OS in a significant way in the TCGA cohort (Figure 5A). Subsequently, by multivariate Cox regression analyses, the risk score was confirmed to be an independent prognostic factor for GC in the TCGA cohort (*p* = 0.002, HR = 1.79, 95% CI: 1.26–2.64) (Figure 5B). Then, the *C*‐index with 95% CI was calculated for the nomogram to quantify its predictive accuracy. The *C*‐index for predicting 1‐year OS is 0.783 with 95% CI: 0.722–0.844; 3‐year OS is 0.752 with 95% CI: 0.713–0.791; and 5‐year OS is 0.726 with 95% CI: 0.690–0.762. With this nomogram, we could forecast 1‐, 3‐, and 5‐year survival probabilities. Next, we established calibration curves for 1, 3, and 5 years to confirm the predictive efficiency of the nomogram (Figure 5D,E), which showed a good consistency between reality and prediction. The calibration slopes of the nomogram using 1000 bootstrap resamplings are listed in Figure S6.

### 3.4. Identification of Four DEG‐Related GC Cell Subpopulations

**Figure 6:**
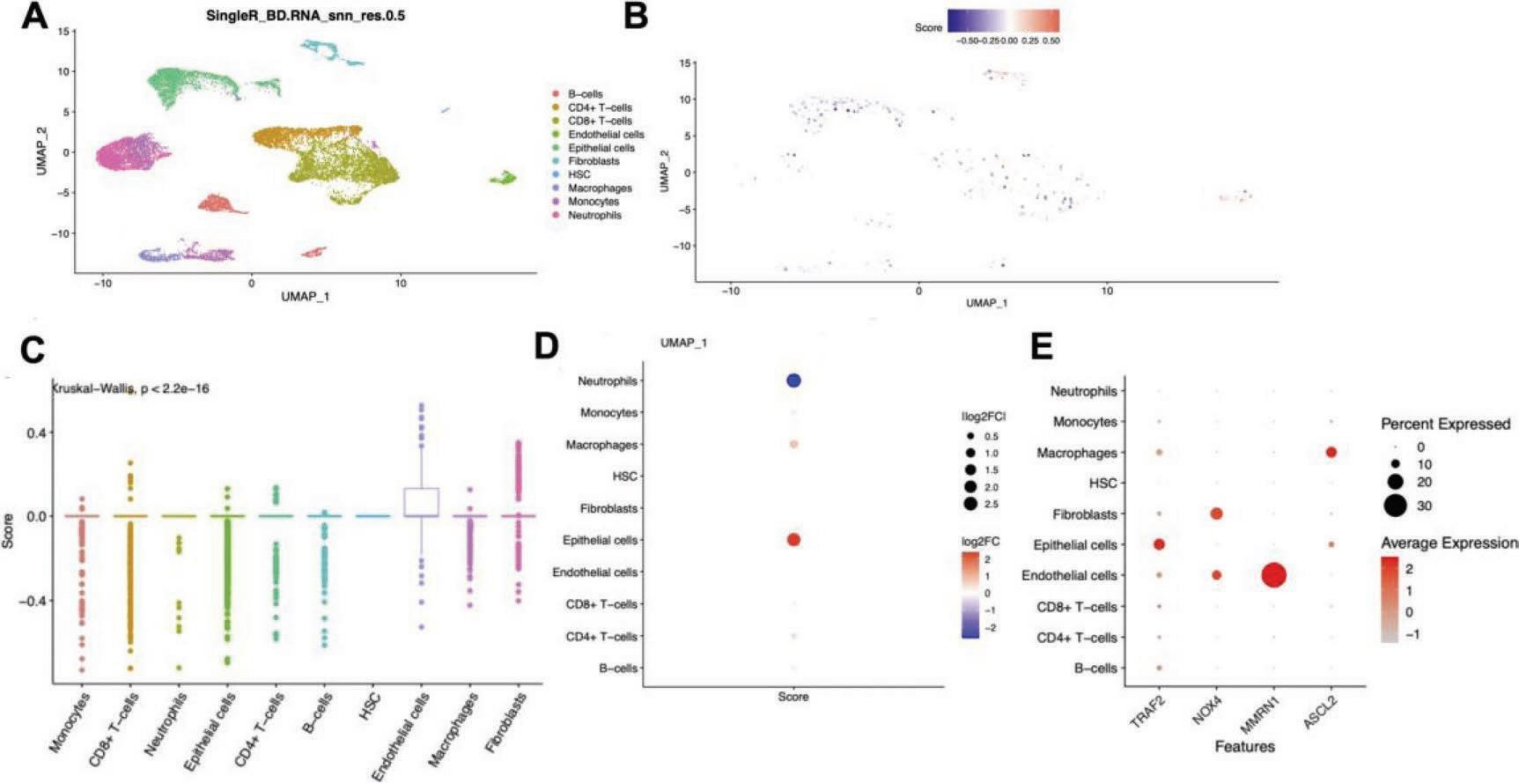
Identification of heterogeneous GC cell subpopulations. (A) First Umap cell subgroups. (B, C) Enrichment for the risk scores varies in different cell subsets. (D) The log2FC scores in different cell subsets. (E) The expression of four DEGs in different cell subsets.

To explore the possible origin of cell subgroups, we analyzed the scRNA‐seq data of GSE163558 to identify the heterogeneous cell subsets. First, we processed the samples, and the cells were annotated into 10 cell clusters by the Umap (Uniform Manifold 154 Approximation and Projection) method (Figure 6A). In several instances, we observed significantly higher ERGRS in endothelial cells, which implies that the four prognosis‐related DEGs possibly mainly originate from endothelial cells (Figure 6B–D). Figure 6E displays epithelial cells with high expression of TRAF2, fibroblasts and endothelial cells with high expression of NOX4, endothelial cells with high expression of MMRN1, and macrophage cells with high expression of ASCL2.

### 3.5. The Function of the Risk Score in Analyzing TME and Forecasting the Response of Immunotherapy

**Figure 7:**
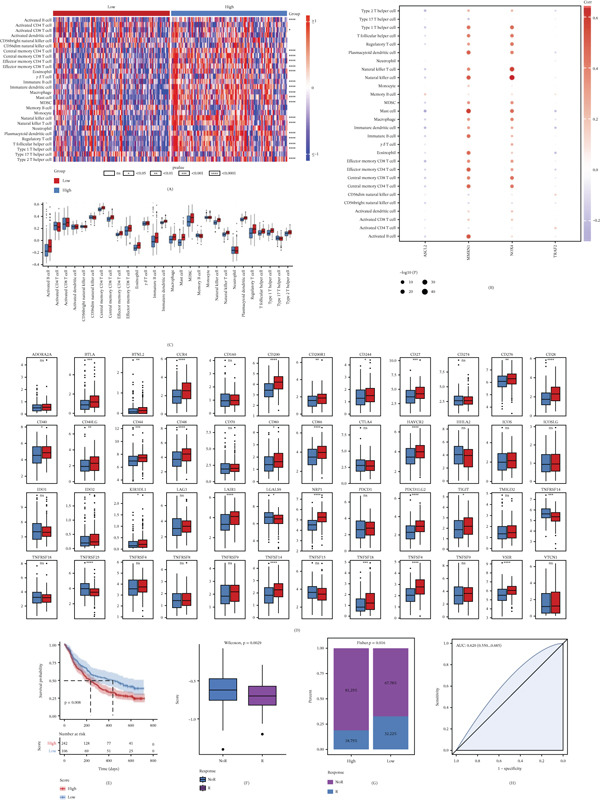
The association between the ERGRS and immune infiltration in the TME. (A) Heat map depicting various immune cells in the high‐ and low‐risk subgroups. (B) Bubble plot of the expression of these four DEGs and immune cells. (C) Differential analysis of the relative infiltration content of 28 types of immune cells between the high‐ and low‐risk subgroups. (D) Difference in the expression of immune checkpoints between the high‐ and low‐risk subgroups. (E) Kaplan–Meier′s curves for patients with high‐risk and low‐risk scores in the IMvigor210 cohort. (F) The comparison of risk scores in groups with different immunotherapeutic treatment response statuses in the IMvigor210 cohort. (G) Treatment response rates of immunotherapy in the high‐ and low‐risk groups in the IMvigor210 cohort (*p* = 0.005). NR represents SD/PD; R represents CR/PR. (H) ROC curves evaluate the predictive accuracy of the risk score in the IMvigor210 cohort.  ^∗^ *p* < 0.05,  ^∗∗^ *p* < 0.01, and  ^∗∗∗^ *p* < 0.001; NS, no significance.

To interrogate the relationship of the ERGRS with immunity statutes, the immune‐infiltrating cell in GC patients was further assessed in the L and H groups based on transcriptomic profiles from TCGA‐STAD. We first explored the expression of each immune‐infiltrating cell across these two groups (Figure 7A). Furthermore, correlations between immune‐infiltrating cells and each one of these four ERGs were analyzed in the TCGA cohorts (Figure 7B). As displayed in Figure 7C, activated B cells, central memory CD4 T cells, central memory CD8 T cells, and immature B cells differed and had significantly higher immune infiltration in the low‐risk groups (*p* < 0.05).

Afterwards, we study whether the ERGRS could predict responses of GC patients to ICIs. The results showed that the expression of 29 genes for immune checkpoints significantly differed between the L and H groups, such as ADORA2A, BTLA, and BTNL2 (Figure 7D). Finally, to further explore the value of ERGRS in predicting the immunotherapy response, the IMvigor210 cohort who received immunotherapy was enrolled in this study for analysis. As Figure 7E shows, patients in the L group have a better prognosis after immunotherapy. At the same time, better responses to immune therapies were observed in L‐group patients (Wilcoxon, *p* = 0.0029, Figure 7F). The objective response rate of immunotherapy was significantly lower in the H group (Fisher′s test, *p* = 0.016; Figure 7G) than that in the L group. The risk score showed an acceptable clinical performance for the selection of immune treatment, as Figure 7H displays (AUC = 0.620).

### 3.6. Evaluation of the Protein Expression of Four ERGs in Exosomes of GC Tissue

**Figure 8:**
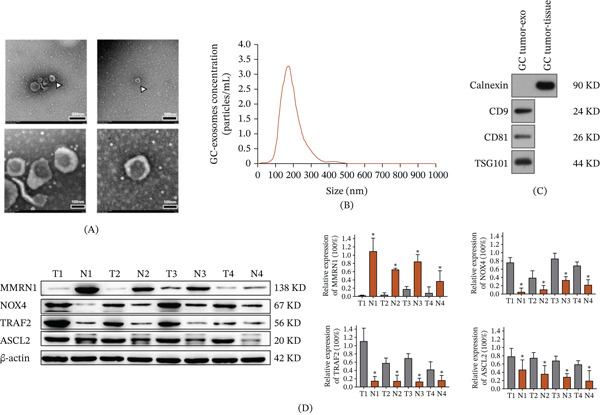
Exosomes in the GC sample were isolated and characterized. (A) Transmission electron microscopy images of exosomes from GC tissues. Scale bar = 100 or 500 nm. (B) The particle size/concentration of exosomes from GC tissues was analyzed by NTA. (C) Western blot analyses of exosomal markers (calnexin, CD9, CD81, and TSG101) of exosomes isolated by ultracentrifugation from GC samples. (D) The protein expression levels of TRAF2, ASCL2, NOX4, and MMRN1 in exosomes from GC tissues.  ^∗^ *p* < 0.05.

To investigate the differences in the protein expression level of these four ERGs in exosomes, we first isolate and characterize exosomes directly from GC clinical samples. Exosomes were extracted by ultracentrifugation at GC tumor concentration of 6.8e + 7 particles/mL. TEM was performed to show the existence of exosomes and visualize the cup‐shaped morphology of purified GC sample exosomes (Figure 8A). NTA was conducted to confirm the size and concentration of exosomes derived from GC tissues. Technology demonstrated that the mean size diameters of GC‐purified exosomes were 91.2 nm (Figure 8B). Moreover, western blot analysis of protein extracted from GC tissue and from GC adjacent normal tissue revealed the presence of specific exosome‐positive markers (CD63, CD81, and TSG101). The exosome‐negative marker calnexin was not absent in exosomes (Figure 8C). Altogether, these results confirmed that the main contents of the purified microvesicles were exosomes.

Furthermore, western blotting was performed to examine TRAF2, ASCL2, NOX4, and MMRN1 protein expression levels in GC tissues and adjacent normal tissues. As Figure 8D displays, NOX4, TRAF2, and ASCL2 protein expression were high in all four GC tissues, and MMRN1 protein expression was low in all four GC tissues, compared with the corresponding adjacent samples.

## 4. Discussion

GC is a common malignant tumor of the digestive tract with a high socioeconomic burden and high morbidity [24]. Considering the widely varying prognostic outcomes of GC and the crucial role of exosomes in tumor progression, our present study for the first time systematically analyzed the ERGs in GC transcriptome data and their association with patients′ prognosis. More importantly, an exosome‐related four‐gene risk signature was construed as a prognosis tool, with acceptable performance in prognostic prediction among the training cohort and validation cohorts. The GC patients in the low‐risk score group showed a better clinical outcome than patients in the high‐risk score group. In addition to this, GC patients with low‐risk scores have a good response to immunotherapy.

In our study, the gene signature was constructed with TRAF2, ASCL2, NOX4, and MMRN1. Important roles of the signature genes identified in this study have been previously reported in multiple cancer subtypes. TRAF2, often in concert with other members of the TRAF protein family, and inactivating mutations of TRAF2 have been associated with tumor development [25]. Yan et al. [26] found that TRAF2 is an oncogenic regulator of Wnt/*β*‐catenin signaling. Wang et al. found that overexpression of SMYD3 and ASCL2 is associated with malignant progression and poor patient outcomes in GC [27, 28]. NOX4 is one of the seven isoforms of the NOX family. Several studies have shown that it can promote cancer occurrence and development in different ways [29, 30]. MMRN1 is a platelet protein and has been identified as a DEG in various cancers [31, 32]. Unfortunately, few studies explored the relationship between prognostic prediction and the four genes in GC. In our study, bioinformatics and experimental results showed that TRAF2, ASCL2, and NOX4 were expressed highly, while MMRN1 was expressed poorly in the GC tissues, which is consistent with the previous studies, indicating that these genes can be noninvasive biomarkers. Besides, the results of gene enrichment suggested that the four genes influencing the development of GC may be related to the biological process of protein kinase activity and homocysteine metabolism, which may provide insights into a new angle of mechanistic research on the cancer‐promoting or tumor‐suppressor function of these four genes.

Driven by advances in sequencing data and bioinformatics, a growing number of studies are now developing models for tumor diagnosis, prognosis, and immune infiltration assessment, aiming to advance the field of precision oncology [33–36]. Many previous studies have attempted to identify and develop prognosis marker models of GC. For example, Wei et al. [37] have constructed a four‐gene prognosis ferroptosis‐related lncRNA signature (AUC = 0.636, training cohort), and Wen et al. [38] used the Cox regression model to construct a 13‐gene prognostic signature (AUC = 0.514, verification cohort). A TGF‐*β*‐associated prognostic model, including five genes, was constructed by Zeng et al. [35] (AUC at 1 year = 0.612, training cohorts). To compare and figure out the advantages of our signature based on the ERG signature, we further analyzed the three‐prognostic gene signature above simultaneously. The results displayed that the 5‐year AUC values of our model are higher than those of other models, indicating that our model uses fewer genes to obtain a more reasonable and reliable prediction result, and may be more suitable for clinical application. Furthermore, to provide an individualized and accurate prediction, a nomogram that incorporated the ERG signature with age and stage was constructed in this study. Compared to the performance of a single indicator, the nomogram contributed more and performed better for survival prediction. The results of the calibration curve and DCA demonstrated that it performed well.

Recent studies have revealed that exosomes are closely related to the TME and are key players in cancer, including GC [39]. Qu et al. first reported the role of exosomes in tumor cell growth and that GC cell–derived exosomes promoted GC cell proliferation by activation of PI3K/Akt and MAPK/ERK pathways [40]. In addition, another important role of GC‐derived exosomes is modulating tumor immunity. Zhang et al. [41] reported that exosomes from GC cells can induce neutrophils to polarize to N2 tumor–associated neutrophils, resulting in prompting GC cell migration. Exosomes can also mediate drug resistance in GC. Ji et al. [42] demonstrated that exosomes secreted by mesenchymal stem cells could induce the resistance of GC cells to 5‐fluorouracil (5‐FU). Nevertheless, the exosome‐related classification of GC is not well defined. Herein, in this study, we figure out four ERGs and construct a signature that can be an independent risk factor, indicating that the underlying mechanism deserves in‐depth analysis.

Immunotherapy is widely applied in various cancers and presents durable antitumor activity against GC therapy [43]. Nevertheless, this therapy still faces many challenges [4]. It has been realized that the TME is complex and tumors may lead to immune evasion by expressing immune checkpoints. Thus, the prediction of the response to ICIs based on immune cell infiltration plays an important role in enhancing the efficacy of immunotherapy [44]. Xu et al. [45] reported that the amplification of TRAF2 was related to the increased expression of PD‐L1 after performing an integrated multiomics analysis of urothelial carcinoma of the bladder. Wu et al. [46] illustrated that ASCL2 was positively correlated to cancer stem cells and tumor immune infiltration and may act as a promising predictor of clinical responsiveness to anti‐PD‐1/PD‐L1 therapy in colon adenocarcinoma. These findings were similar to the results in the current study. We found that the risk score of the gene signature was negatively related to patients′ response to immunotherapy. The immune infiltration analysis showed that the level of risk score was correlated positively with the expression of activated B cells, activated CD8+ T cells, and macrophages. Collectively, these results indicate that the development of the risk score is negatively correlated with the prognosis of GC patients. However, the IMvigor210 cohort consists of urothelial carcinoma patients, and using this cohort to predict immunotherapy response in GC is another limitation. Hence, our study is a preliminary, cross‐cancer extrapolation.

Several limitations of this study should be acknowledged. First, the findings are primarily derived from bioinformatic analyses and lack functional experiments (e.g., knockdown/overexpression in GC cell lines and exosome functional assays) to demonstrate how the ERGs mechanistically influence GC progression or exosome‐mediated communication. Second, other well‐established prognostic factors for GC—such as family history and serum tumor markers—were not incorporated, which could potentially improve the predictive accuracy of the model. Finally, the sample size of GC tissues was limited, and the clinical utility of the risk score in predicting prognosis and immunotherapy response warrants further comprehensive investigation, including integration with metabolomic and proteomic data.

## 5. Conclusions

In summary, we developed a novel ERGRS that predicts prognosis in patients with GC. A high risk score based on this signature serves as an independent prognostic factor in GC. By integrating the signature with age and tumor stage, we further constructed a nomogram that effectively predicts survival outcomes in GC patients.

## Author Contributions

Study conception and design and data collection: Xiulan Peng, Qunru Jiao, and Yingyun Guo. Statistical analysis: Rui Zhang and Longshu Zhou. Manuscript draft, review, and editing: Yingyun Guo, Xiulan Peng, and Longshu Zhou. Qunru Jiao, Rui Zhang, Yingyun Guo, and Xiulan Peng contributed equally to this work.

## Funding

No funding was received for this manuscript.

## Ethics Statement

The study had been approved by the Ethics Committee of Renmin Hospital of Wuhan University in Hubei Province (No. WDRY2021‐K002).

## Conflicts of Interest

The authors declare no conflicts of interest.

## Supporting information


**Supporting Information** Additional supporting information can be found online in the Supporting Information section. Figure S1: (A) Volcano plot and (B) heat map of 300 differentially expressed genes (DEGs) from the exoRbase dataset. (C) Volcano plot and (D) heat map of 2663 genes identified from the cohort of TCGA‐STAD. Figure S2: Bar plot of GO enrichment analysis for DEGs: (A) biological process, (B) cellular components, and (C) molecular functions, respectively. (D) Bar plot of KEGG enrichment analysis for DEGs. Figure S3: (A) The mutation rate of MMRNN1, TRAF2, and NOX4 through SNV analysis was 2%–4%. (B) The DEG CNV showed the mutation rate of TRAF2 (16%), ASCL2 (7%), NOX4 (6%), and MMRN1 (5%). Figure S4: (A) Forest plots of univariate and (B) multivariate Cox regression, and TRAF2, ASCL2, NOX4, and MMRN1 screened as high‐risk genes. Figure S5: The survival analysis comparing the DFI event in the H and L groups showed similar results to OS. (A) Distribution of risk scores and survival status. (B) Expression of four prognostic genes. (C) Kaplan–Meier′s analysis was used to compare the DFI time of patients in the high‐risk and low‐risk groups. (D) Time‐dependent ROC curves of the ERGRS for predicting DFI at 1, 3, and 5 years. Figure S6: Calibration slopes using 1000 bootstrap resamplings. Table S1: Baseline characteristics of patients in the TCGA‐STAD cohort. Table S2: List of 34 exosome‐related OCGs in GC via Venn analysis.

## Data Availability

The data that support the findings of this study are available on request from the corresponding authors.
