# Supplementary material for: Exosome‐Related Gene Signature Predicts Prognosis and Immunotherapy in Gastric Cancer
Source: Int J Genomics. 2026 Jun 19;2026:1884334. doi: 10.1155/ijog/1884334 (PMC13280462; doi:10.1155/ijog/1884334)
Supplement: Supplementary file 1 — Supporting Information Additional supporting information can be found online in the Supporting Information section. Figure S1: (A) Volcano plot and (B) heat map of 300 differentially expressed genes (DEGs) from the exoRbase dataset. (C) Volcano plot and (D) heat map of 2663 genes identified from the cohort of TCGA‐STAD. Figure S2: Bar plot of GO enrichment analysis for DEGs: (A) biological process, (B) cellular components, and (C) molecular functions, respectively. (D) Bar plot of KEGG enrichment analysis for DEGs. Figure S3: (A) The mutation rate of MMRNN1, TRAF2, and NOX4 through SNV analysis was 2%–4%. (B) The DEG CNV showed the mutation rate of TRAF2 (16%), ASCL2 (7%), NOX4 (6%), and MMRN1 (5%). Figure S4: (A) Forest plots of univariate and (B) multivariate Cox regression, and TRAF2, ASCL2, NOX4, and MMRN1 screened as high‐risk genes. Figure S5: The survival analysis comparing the DFI event in the H and L groups showed similar results to OS. (A) Distribution of risk scores and survival status. (B) Expression of four prognostic genes. (C) Kaplan–Meier′s analysis was used to compare the DFI time of patients in the high‐risk and low‐risk groups. (D) Time‐dependent ROC curves of the ERGRS for predicting DFI at 1, 3, and 5 years. Figure S6: Calibration slopes using 1000 bootstrap resamplings. Table S1: Baseline characteristics of patients in the TCGA‐STAD cohort. Table S2: List of 34 exosome‐related OCGs in GC via Venn analysis. [file IJOG-2026-1884334-s001.pdf]

Exosomes-related gene signature predicts prognosis and immunotherapy in gastric cancer

Qunru Jiao[1*], Rui Zhang[2*], Yingyun Guo[3*], Xiulan Peng[4*], Longshu Zhou[5],

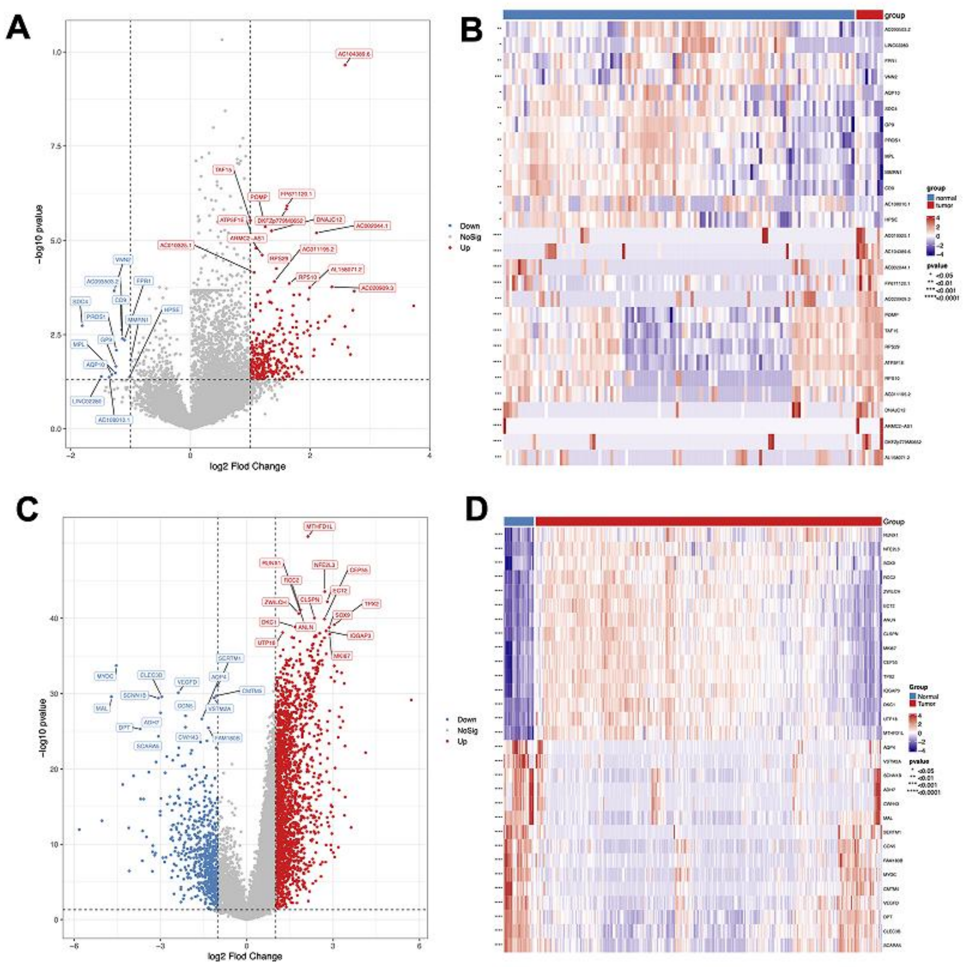

Figure S1. Volcano plot **(A)** and heat map **(B)** of 300 differentially expressed genes (DEGs) from the exoRbase dataset. Volcano plot **(C)** and heat map **(D)** of 2663 genes identified from cohort of TCGA-STAD.

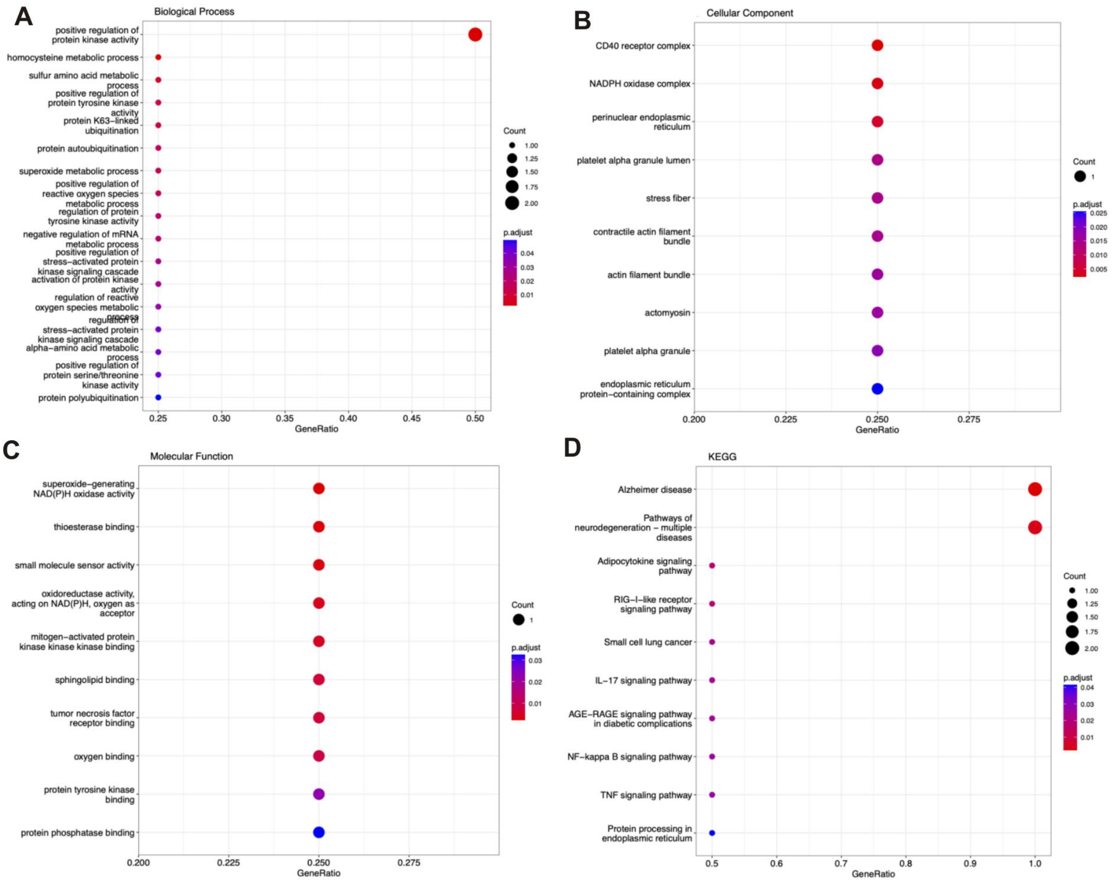

Figure S2. barplot of GO enrichment analysis for DEGs, (**A**) biological process, (**B**) cellular components, and (**C**) molecular functions, respectively. (**D**) bar plot of KEGG enrichment analysis for DEGs.

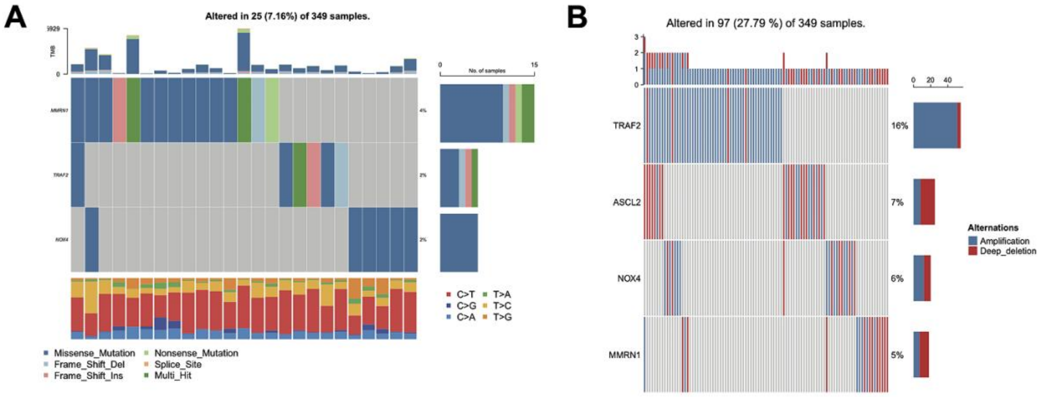

Figure S3. (A) The mutation rate of *MMRNN1*, *TRAF2*, and *NOX4* through SNV analysis was 2%-4%. (**B**) The DEEGs CNV showed that the mutation rate of *TRAF2*（16%）, *ASCL2*（7%）, *NOX4*（6%）, *MMRN1*（5%）.

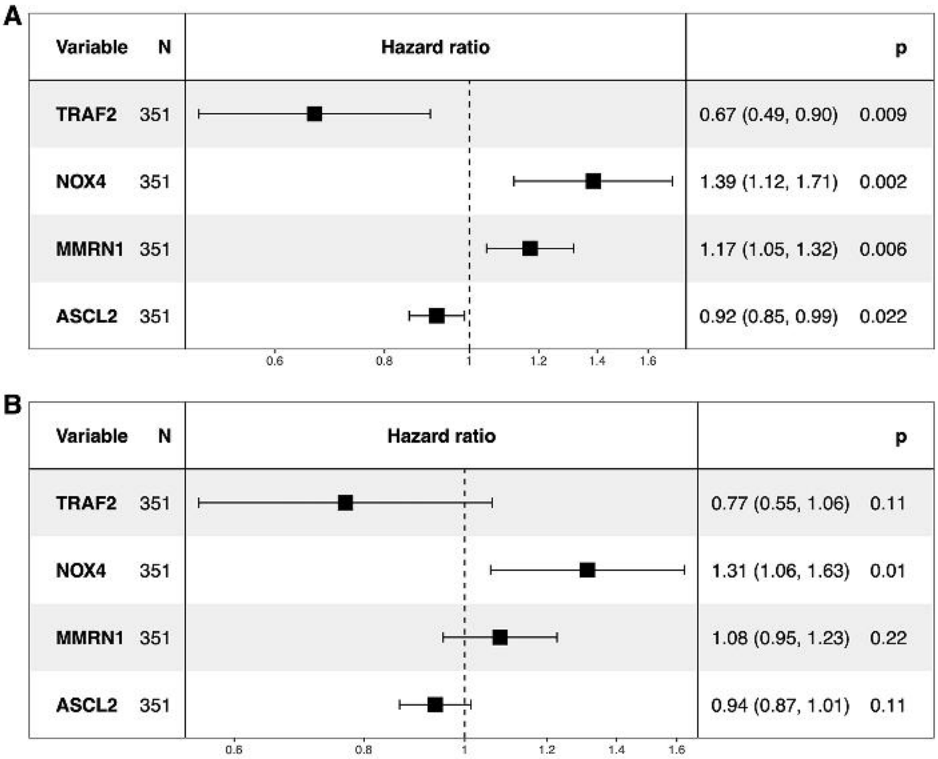

Figure S4 Forest plots of univariate (**A**) and multivariate (**B**) Cox regression, and*TRAF2, ASCL2, NOX4, MMRN1* were screened as high-risk genes.

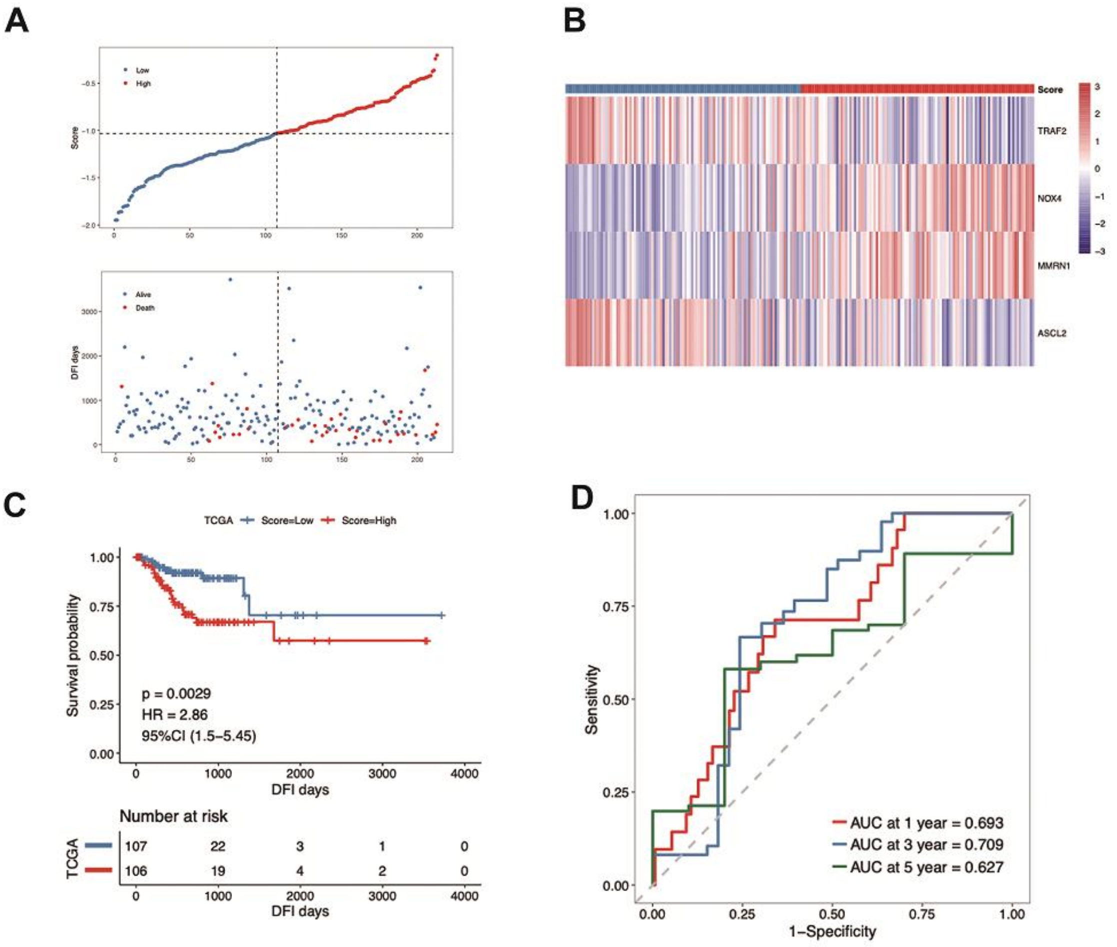

Figure S5 The survival analysis comparing the DFI event in the H- and L-groups showed similar results to OS. (**A**) Distribution of risk scores and survival status. (**B**) Expression of four prognostic genes. (**C**) Kaplan-Meier analysis was used to compare the DFI time of patients in the high-risk and low-risk groups. (**D**) Time-dependent ROC curves of the ERGRS for predicting DFI at 1-, 3-, and 5- years.

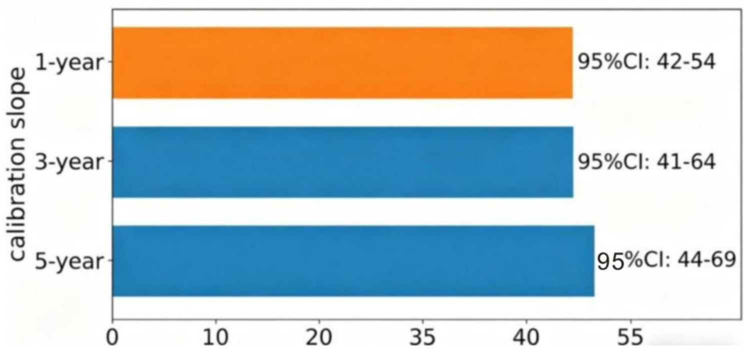

Figure S6. Calbration slopes using 1000 bootstrap resamplings.

Table S1 Baseline characteristics of patients in the TCGA-STAD cohort.

**Variables**	**Overall** (N=351)
Age	
<60	108 (30.8%)
>=60	240 (68.4%)
Missing	3 (0.9%)
Gender	
FEMALE	124 (35.3%)
MALE	227 (64.7%)
Stage2	
I	48 (13.7%)
II	109 (31.1%)
III	145 (41.3%)
IV	34 (9.7%)
Missing	15 (4.3%)
Grade	
G1/G2	135 (38.5%)
G3/G4	207 (59.0%)
Missing	9 (2.6%)
h_pylori_infection	
No	142 (40.5%)
Yes	18 (5.1%)
Missing	191 (54.4%)

Table S2 List of 34 exosomes-related OCGs in GC via Venn analysis.

number	Exosome-related gene
1	CENPL
2	TRAF2
3	CLEC3B
4	CENPA
5	POLR1C
6	ZNF485
7	PAQR4
8	HSPE1
9	DTYMK
10	ARPC1B
11	POLR3K
12	AQP10
13	TMEM100
14	CKLF
15	SOSTDC1
16	BCL2L12
17	GPR4
18	HPSE
19	NOX4
20	SLC35F2
21	MMRN1
22	TCEAL2
23	COX7A1
24	SNRPN
25	DNAJB5
26	IDO1
27	CCL3L1
28	ASCL2
29	CCL4L2
30	HOXB6
31	OSM
32	ETV7
33	LAIR2
34	TSPAN7
